# Supplementary material for: Anesthetics drug wastage and preventive strategies: Systematic review
Source: PLoS One. 2024 Jul 17;19(7):e0306933. doi: 10.1371/journal.pone.0306933 (PMC11253927; doi:10.1371/journal.pone.0306933)
Supplement: S1 File — (PDF) [file pone.0306933.s001.pdf]

Supporting information

Preferred Reporting Items for Systematic Reviews and Meta-Analyses: The PRISMA Statement

Section/topic	#	Checklist item
**TITLE**		
Title	1	Anaesthetics drug wastage and preventive strategies: systematic review
**ABSTRACT**		
Structured summary	2	Background: Surgical Patients and hospitals are now facing financial strain d as a result of the development of new anesthetic drugs, equipment, and hospital's pharmacy budget is currently allocated to anesthetic drug exp anesthesia practice is a widespread hidden source of healthcare waste that lea as well as poor operating room efficiency. On the other hand, despite the fa vast majority of cases, it is well described that drug wastage is routinely obs countries where the consequence significantly affect both hospitals and patien<br><br>Methods: This review aims to revise the prevalence of anesthetics drug systematically formulate and describe the preventive strategies. Relevant pub systematic search on data bases including the Google scholar, Medline (Pu Embase. In addition papers were detected and then selected through the Systematic reviews and Meta-Analyses (PRISMA) criteria guidelines and criteria. Using the predetermined terms and date, from the searching data ba

identified. Based on the screening criteria, 16 papers were considered to be el
review. In addition the Joanna Briggs Institute (JBI) Manual for Evid
evaluating the quality of selected articles. This study is registered
CRD42024497044.

Results: Of the fifteen publications of ten different nations that considered sui
them addressed the waste of inhalational anesthetics. In more than half of elig
frequently wasted drug that contributed for increased financial loss by dr
significant factor contributing to the waste of intravenous and inhalational a
multidrug vials following their use for a single patient and high fresh gas flow

INTRODUCTION Due to the advancement of novel anesthetic drugs, equipment, and procedures, anesthetic costs are
burden for both patients and hospitals[1]. Particularly, the considerably increased cost of anesthetic medicines have a sig
in developing countries[2]. According to statistics, operation room (OR) account for 40% of total hospital expenditure, ye
for 5-15% of a hospital's pharmacy budge[1, 3]. Possible changes in the individual response of patients, unused drug
because of sterility concerns make some amount of drug wastage unavoidable in anesthesia [2, 4, 5].

Numerous pieces of data indicate these anesthetic medication waste may occur at any time of the perioperative period, yet
an emergency[6, 7]. Wastage is also described to be particularly prevalent with intravenous drugs due to the fact that th
anesthetic agents, are packaged in specific amounts in ampoules or vials[4, 8]. These drugs can only be withdrawn after
the rubber stopper is penetrated by the hypodermic needle. Thus they need to be used within a specific period, due to con
and contamination. Partially used ampoules/syringes or loaded but unused syringes are usually discarded after the end of a

Additionally, it is emphasized that a significant portion of the inhalation agents are not absorbed by the patient, but rather
through the waste gas scavenging mechanism[10, 11]. When computing medication waste in the operating room, volatile

than 20% of anesthesia expenses must also be taken into consideration. This is especially important now that newer and sevoflurane, are in clinical use[12, 13].

On the other hand, such wasted drugs have been shown to contribute significantly to the cost of intra operative anesthesia In addition to the hospital's financial costs, discarded medications like propofol contaminate the environment and consequences. Moreover, it can increase occupational hazards for health care and sanitary workers and cause environm reduction strategies have gained considerable significance due to rising anesthetic drug prices, health care costs, parti Inferring that decreasing drug waste is a key area for lowering anesthetic costs without compromising the quality of care d

Although potential sources of anesthesia-related waste are not limited to drugs, for the purposes of current review, we fo waste. The first reason is that it has been claimed that a hidden source of healthcare waste[5]. Another explanation is that, in drug costs, anesthetic drug wastage directly contributes to anesthetic drug shortages as well as poor hospital efficie Despite the fact that there are several original studies and a few brief reports on the topic, to the best of our knowl evaluation that examined most recent publications for the waste of anesthetic drugs and their preventative measures t enhancing the hospital's anesthetic clinical practice in a safe and cost-effective manner.

Rationale	3	Although potential sources of anesthesia-related waste are not limited to dru review, we focused on anesthesia-related drug waste. The first reason is th hidden source of healthcare waste[5]. Another explanation is that, with the cu costs, anesthetic drug wastage directly contributes to anesthetic drug short efficiency in surgical patient care[17]. Despite the fact that there are several reports on the topic, to the best of our knowledge, this is the first systematic recent publications for the waste of anesthetic drugs and their preventativ them, therefore enhancing the hospital's anesthetic clinical practice in a safe a
Objectives	4	The primary objective of this review was to determine the global prevalen clinical practice of anesthesia. Thus, this review aimed to answer the followin

		I. What is the prevalence of commonly used anaesthetics drugs?<br>II. What are the causes of intravenous and inhalational agent wastage?<br>III. What are the possible preventive strategies for anaesthetics drug wasta
**METHODS**		
Protocol and registration	5	The protocol was registered in the International Prospective Regis (CRD42024497044).
Eligibility criteria	6	Types of studies: We include all published studies on prevalence and cause also have included randomised controlled trials, but we found none. Papers w through the PRISMA (Preferred Reporting Items for Systematic reviews guidelines and the inclusion and exclusion criteria [18]. Only peer-reviewed written in English, published after 2010 and which presented the magnitud drug wastage in OR considered in this review. The publications that were e (that is, unrelated to the prevalence, significance, or predicting factors of solely addressed other anesthetic wastes (such as waste pertaining to operati evaluation of only utilized anesthetics drugs) were then excluded fro publications that solely detailed anesthetic drug waste in settings other th intensive care units, emergency rooms, catheterization suite, ambulatory surg care unit) were disregarded.
Information sources	7	We conducted electronic searches for eligible studies within each of the follo 20 December 2023).<br>The Google scholar,<br>Medline (Pubmed),

		Cochrane Library and Embase
Search	8	Databases were searched using the following terms: anesthesia, drug waste, anesthet factors, intravenous anesthetics, inhalational anesthetics, operating room efficiency, room, surgery, preventive strategies, and waste in the operating room. Addition phrases were combined with Boolean operators (AND, OR) for additional searches.
Study selection	9	Two researchers (TY and BA) independently reviewed titles and abstracts of the inconsistencies until consensus was obtained. Then, in pairs, the researchers in abstracts of all articles retrieved. In case of disagreement, consensus on which articl by discussion. If necessary, the third researcher (MF) was consulted to make the fin (BA and TY) independently screened full-text articles for inclusion. Again, in case reached on inclusion or exclusion by discussion and if necessary, the third researche
Data collection process	10	We developed a data extraction form to extract study characteristics and the tested by two study team members using five randomly selected studies. Re to extract study details. A third reviewer reviewed data extraction, and resolv
Data items	11	We reported measures of outcomes from included studies that were adjust frequency and potential causes of anaesthetics medication wastage.
Risk of bias in individual studies	12	the Joanna Briggs Institute (JBI) Manual for Evidence Synthesis were use selected articles[19].
Summary measures	13	N/A, Because all of the included research are observational in design.
Synthesis of results	14	The result of studies was examined and summarized consistently and coher drug studied on.

Section/topic	#	Checklist item
Risk of bias across studies	15	N/A, because we don't perform meta-analysis here.
Additional analyses	16	N/A, because we don't perform meta-analysis here.
**RESULTS**		
Study selection	17	By using the search parameters, 504 papers in total were found. Following the scree of inclusion and exclusion criteria, 22 of these were deemed appropriate for full-tex were included in the final review. Table 2
Study characteristics	18	The studies that were analyzed covered the prevalence as well as possible rea of medication wastage during anesthesia practice. The selected articles are fr including Ethiopia and Nigeria. Eleven research looked at how much medica wasted, and how much money was lost due to avoidable drug waste [2, 8, incorporates material waste into its primary outcome, medication waste in majority of studies on intravenous anesthetic drug loss consider drug wastage into syringes but are either partially or completely unused or discarded at the [6-8, 11, 25]..
Risk of bias within studies	19	Not applicable
Results of individual studies	20	Not applicable
Synthesis of results	21	Not applicable
Risk of bias across studies	22	Not applicable

Additional analysis	23	Not applicable
**DISCUSSION**		
Summary of evidence	24	The primary finding of the publications included in this systematic review waste of anesthetic drugs in the operating room (OR), which has been relatec financial efficiency. It also negatively affects the environment because im contaminate water supplies and possibly cause health risks to humans (11).<br>Propofol is the expensive drug with several advantages including faster rec nausea-vomiting and shortened stay in post anesthesia care unit (PACU) environmental implications as it does not degrade in nature, accumulates in b life(4). Despite these benefits and its potential environmental impact upon di of numerous studies, propofol was shown to be the most wasted medicine ac drug waste measured in milliliters(4, 30). Due to partially unused after loadir vial (MDV) disposal following a single patient's use, propofol was detern wasted drug in more than half of the reviewed articles (4, 21, 24).<br>An observational study of 356 procedures conducted by Amucheazi Adaobi was one of the most commonly wasted drugs, accounting for more than 50% Similarly, an Indian study that examined the amount and financial implicati syringes/vials after completion of each case found that the cost of wasted ane the study takes in 46.57% of the entire cost of drugs loaded (Rs. 16,044.01 waste contributed the most to overall waste, accounting for 56.27% (11).<br>In terms of analgesics, the findings of three studies revealed a significant amo including narcotics, during anesthesia practice. A study by Hailu et al fi

tramadol, diclofenac, and fentanyl were among the most loaded in syringes b
than 273 Ethiopian birr waste during the two week study period(6). Morphin
lost drugs due to not being utilized after loading into syringe, accounting fo
intravenous anesthetic drug wastage, respectively, according to a related
analysis of annual narcotic wastage in Saudi Arabia revealed that the h
observed for Morphine 10 mg formulations (1956 ampoules), and the autho
prefilled syringes with calculated doses for a specific patient provided b
significant waste reduction and cost savings(6).

Despite the fact that wasted inhalational anesthetic gases are expensive an
environment, there have been limited studies on the subject. Likewise, onl
considered inhalational agent waste (12, 24). This could be because inhalatio
liquid formulations but are administered as vapors; as a result, it is more
much inhalation agent is wasted (31-33). For prospective study of 258 adult
divided maintenance phase of anesthesia into low (FGF $\leq$2 L/min) and h
sevoflurane and isoflurane with a targeted MAC of 0.8 1.2(24). The result
mean cost in high flow groups was higher than that of low flow groups a
anesthesia with isoflurane is more cost effective as compared to high flow tec

Moreover, significant drug wastage has also been documented followi
medications (atropine, glycopylorate, adrenaline, epinephrine, phenyleph
anesthetics (bupivacaine, lignocaine), neuromuscular medications (atracuriu
and vecuronium), and other medications (neostigmine, diazepam, midazolam)

Preventive strategies for drug wastage in operating theater

Waste-reduction measures in healthcare are becoming more and more

		consideration in an era where hospital and patient costs are rising (20, 34). Ev cannot be restricted to save expenses and so compromise patient care, the e would be advantageous in reducing drug waste (2, 20, 35). As a result, the are discussed after a thorough review of all relevant studies published after 20<br>Split dose<br>If the contents of an ampoule are likely to be used on more than one patien drugs into several syringes, or “split doses”(8). To ensure an efficient distrib weight and according to the need and demand of each case, prefilled syring remaining amount in multidose vials can be used to provide prefilled syringes However, it is not necessarily extended to the preparation of other drugs, suc used during the case but are discarded after the procedure(4). On the other h dose is considered, special attention should be given to avoid excessive volu were one of the frequent sources of drug waste that multiple research have hig<br>Propofol<br>Disposing of the multidrug vials (MDV) after using them for a single patient cause of propofol waste. Similarly, findings have revealed that opening induction and filling 50 ml syringe pumps for the maintenance of a short cas of this wastage. Additionally, because it is advised to discard vials after 6 ho vials on days with a short OR case list, one major case, or cases under region significant amount of drug left in the vial or syringe to be wasted(4, 20, 26).<br>Given the high cost, environmental impact, and short shelf-life in the syringe optimize its use, ideally, by preparing appropriate dose of it when only actu

that no patient needed more than 20 ml of propofol to get induction of anesth
prevent medication waste from leftover syringe, it would be recommended t
range for induction (2.5 mg/kg), which would be less than 20 ml for a patien
70 kg(20).

Additionally, purchasing 20 ml vials would be another better option f
management. Even though smaller vials have a greater unit price than 50 m
reduce actual financial loss by lowering overall wastage. As a result, selec
communicating about the anesthetic plan in advance may help to reduce wa
vial of propofol on hand in the operating room also helpful to prevent wastag
volume of propofol into syringes for cases including regional anesthesia and
36).

Inhalational anaesthetics agent (IAA)

It is well known that there is potential for cost reduction in the field of anesth
inhalation agents, as they account for a significant portion (20–25%) of the c
drugs(3, 10). Scientific reports indicate a clear relationship between the fresh
of inhaled anesthetic agent wastage (37). A high flow rate reduces the amount
is rebreathed and increases the amount of vaporized inhaled anesthetic
anesthetic gas vaporized exceeds what partitions from the gas phase into the
anesthetic gas ends up being vented into the atmosphere through the waste ga
Using a low fresh gas flow rate, on the other hand, maximizes rebreathing
reduces the amount of anesthetic gas wasted by venting into the atmosphere
over a 2-hour anesthetic period with 4.4 l/min vs 1.0 l/min fresh gas flows wa

		0.23, respectively, in the study. This indicates that the patient absorbs just 7 remaining 93% going waste at a flow rate of 4.4 l/min (37).<br>To reduce wastage of excess agents, a study was proposed to evaluate t notification by Smart Anesthesia Messenger system that remind the anesth within the limits recommended by food and Drug Administration of the co conducted on one baseline and three interventions phases of surgical proc either of sevoflurane, isoflurane or disflorane. The authors reported that they l of desflurane, and 0.8 l isoflurane per month, translating to an annual sa concluded that real-time notification is an effective way to reduce inhalation a excess FGFs (12).<br>Neuromuscular blocking drugs (NMBD)<br>According to Kaniyil (2017), Vecuronium and rocuronium, in particular, wer most often wasted medication, accounting for 35.21% of the drug waste in OI by loading the medication appropriately for each case (based on the kg of bod medication that remains in the multidose vial to be used in subsequent cases addition, to reduce waste, it is crucial to make thoughtful decisions about the in each situation, including continuously monitor if a neuromuscular blocker and load the medication in accordance with the requirements of the patient(2)<br>The medication doses for each case be calculated based on the patient's weigh that drug for the procedure, and that the dose be shown on the machine prior case(38). It would help in determining the patient's dosage requirements and e reducing waste.

Local anesthetics

Numerous prospective drug wastage and cost analysis of wastage was observ bupivacaine for a single patient. The majority of wastage related to loading o mainly for prevention of pain of propofol injection is reported. Lignocaine m 1–2 ml may be loaded in the syringe and given prior to propofol injection i lignocaine multidose vials on/near the anesthetic machine, in case of need, v extent pdf 2.

As a result, using 2 ml instead of 4 ml of bupivacaine ampules could reduce residue in vial or syringe waste and ease the financial burden on patients and mix 1-2 ml of lignocaine with propofol or to load in the syringe and administ rather than loading 5 ml in each case. Additionally, ensuring that lignocai accessible on or next to the anesthetic machine would significantly reduce wa

Emergency (resuscitation) drugs

By considering that time in emergency scenario to prepare the drugs in a sy increase dilution error, in many operating set up it is a standard practice induction of anesthesia. As a result, anesthesia care providers commonly dra resuscitation drugs including ephedrine, phenyleprine, and atropine prophyla drugs are not always needed and because of concerns about infection control, used for one patient are usually not administered to subsequent patients. So syringe doses may be discarded at the end of a clinical workday(20, 22).

A retrospective study from France, to determine the number of patients give 27 705 operations shown that the rate of wastage of atropine and ephedrine, t to 67% and up to 91% respectively(28). Similarly, related study also reveal

epinephrine and atropine, are almost always prepared, but rarely used ar
wasted(38). In settings where the anesthesiologist works alone, or where ane
trained, the practice of loading one ampule of atropine, adrenaline and ephed
surgery may seem relevant in anticipated cases (11, 20, 38). It may be
resuscitation will be required by taking into account the patient's vascular
block, and adequacy of fluid preload prior to neuraxial anesthesia.

Additionally, It is also possible to make sure that it is easily accessible intrac
load medication just when necessary, Otherwise routine Preoperative preparat
is rarely justified and a safer approach would be to store emergency drug
syringe on or close to the anesthetic machine so that it is ready to load and u
hand, by loading 15 mg of mephentermine sulfate in situations when hypoter
30 mg in every case, mephentermine sulphate waste can be reduced by almost

Furthermore, establishing an emergency drug tray attached to workstation
labeled, and ensuring that it is immediately available on or near the anesthesia
strategy to prevent unwanted loading of all drugs and ensure availability in ca

Improve provider’s awareness towards anesthetic drug wastage

Every healthcare provider must consider the financial effects of the decision
and optimize practice patterns (2). Despite this growing body of knowledge,
be a high priority for the average anesthesia practitioner, and cost awareness
waste may require interventional education in the form of lectures th
recommended practices, as well as the placement of posters in operating
rooms, and recovery rooms (22).

Aside from posters, the drug prices on the anesthesia machine are anoth

		financial constraints. Individual hospital efforts to raise awareness about red<br>expensive medications responsibly can be effective. Furthermore, education p<br>anesthetic drug waste and drug wastage strategies, are recommended (11).<br>AUDIT<br>Finally, a regular review and audit for the storage, use, and ease of rap<br>anesthetic drugs should be carried out in order to better understand th<br>recommendations and determine whether additional adjustments are needed f<br>(11, 20). Incentives for practitioners and OR teams who reduced waste w<br>enforce waste-reducing practices and promote competition. The develop<br>discussions that focus on reducing the costs related to the waste of anest<br>important. Furthermore, there should be a properly functioning incident rep<br>documentation, evaluation, and resolution of challenges related to anesthetic d
Limitations	25	Even though this systematic review includes recent publications from all over the w<br>current trends of anesthetic medication waste in operating rooms, the number of a<br>been limited, particularly when it comes to inhalational agents. Additionally, waste<br>other sources are not addressed. Furthermore, preventive measures are broader in r<br>patient characteristics or surgical techniques into account. Thus it is advised that the<br>included in future research investigations.
Conclusions	26	In conclusion, this review showed the increasing rate of medication wastage in ope<br>collective and standardized effort is needed to improve the efficient utilization of<br>developing countries.
**FUNDING**		

Funding	27	This systematic review received no funding
