# Supplementary material for: Anesthetics drug wastage and preventive strategies: Systematic review
Source: PLoS One. 2024 Jul 17;19(7):e0306933. doi: 10.1371/journal.pone.0306933 (PMC11253927; doi:10.1371/journal.pone.0306933)
Supplement: Attachment — Submitted filename: Authors response@ plos.docx [file pone.0306933.s002.docx]

Manuscript PONE-D-24-00988

Response to Reviewers

Dear Dr. Shweta, Thank you for giving us the opportunity to submit a revised draft of the manuscript “Anesthetics drug wastage and preventive strategies: systematic review” for publication in the Journal of PLOS ONE. We value the time and effort that you and the reviewers have dedicated to providing feedback on our manuscript and are grateful for the insightful comments and valuable improvements to our paper. We have incorporated most of the suggestions made by the reviewers. Those changes are highlighted within the manuscript.

Editor’s comment

1. Please ensure that your manuscript meets PLOS ONE's style requirements, including those for file naming.

Author response: Thank you, and we corrected based on the given guidelines, and it is highlighted.

2. The manuscript requires thorough grammatical revision as the language currently has many errors.

Author response: Thank you, and we have gone over the entire article again and fixed any grammatical issues. However, it is difficult to highlight the fixed grammatical errors because they appear throughout the manuscript.

3. A more detailed environmental impact of inhalation agents and narcotics waste if included in the studies could be mentioned.

Author response: thank you again so much, and we have discussed the environmental impact of both inhalation agents and narcotics wastes.

4. A mention of billing challenges in multi dose vials and sterility concerns for drugs like propofol would be pertinent.

Author response: Thank you, and we explained the sterility concern of extending the strategy of split doses of multi dose vial for propofol.

5. Please review your reference list to ensure that it is complete and correct. If you have cited papers that have been retracted, please include the rationale for doing so in the manuscript text, or remove these references and replace them with relevant current references.

Author response: revised and it is correctly cited

Reviewers' Comments to the Authors:

Reviewer 1

Well done manuscript on a topic that is critical especially in developing countries. I appreciate their critical appraisal of all papers.

Author response: Thank you!

1. I do request they double check their grammar and types as there are several throughout the paper.

Author response: we appreciate you bringing this to our attention. The reviewer is correct, and we have gone over the entire article again and fixed any grammatical issues.

Reviewer 2

Overall, although lacking potential novelty, the paper is sound and easy to read, the topic is interesting.

Author response: Thank you!

However, there are some minors should be addressed before publication.
1. In the title, the authors proposed the preventive strategies to avoid anesthetic drug wastage, like split dose, is there any limitations using this approach? Like splitting dose could also cause wastage?

Author response: As suggested by the reviewer, we incorporated the general limitations of implementing split doses in our response, which we greatly appreciate.

2. Propofol and inhalational anesthetics were suggested to be the most waste drugs in OR. However, is there any difference between inhalational drugs? Like isoflurane or sevoflurane?

Author response: Thank you! But even we conducted an unrestricted search to determine whether the incidence of drug waste varies among inhalational agents for the purposes of this review, but we were unable to uncover any information on the subject.
