# Supplementary material for: Anesthetics drug wastage and preventive strategies: Systematic review
Source: PLoS One. 2024 Jul 17;19(7):e0306933. doi: 10.1371/journal.pone.0306933 (PMC11253927; doi:10.1371/journal.pone.0306933)
Supplement: Attachment — Submitted filename: aouthors response for reviewer comment plosone.docx [file pone.0306933.s003.docx]

Manuscript ID: [PONE-D-24-00988R1] - [EMID:4a1c4deda2223cae]

Response to Reviewers

Dear editor

Thank you again for the chance to submit a revised version of our manuscript titled "Anesthetics Drug Waste and Preventive Strategies: A Systematic Review" for consideration in the Journal of PLOS One. We have incorporated the majority of the reviewer's suggestions, and these revisions are clearly indicated (highlighted) within the manuscript. Sincerely,

[Meseret Firde]

Editor’s comment

After obtaining further recommendations, I encourage you to address the comments raised by Reviewer 4.

A concern raised previously by myself as well is the practice of multiple-use vials and the possibility of contamination and billing issues. A more balanced approach would be to suggest practice changes that would not raise concerns raised by reviewer 4 and myself. Since there is not much evidence available to support these practices, they can be included as limitations of the study.

Author response: Thank you, and we have thoroughly addressed this issue in both the subsection on 'split dose' and in the limitations of the review.

Reviewers' Comments to the Authors:

Reviewer 4

This is a potentially interesting study suitable for the journal's mission and readership.

Author response: Thank you!

1. Although I do appreciate the systematic review performed by the authors, there isn't a lot of literature on the topic. I am not convinced the existing literature is actually helping the authors address the topic other than by highlighting the gaps in the literature.

Author response: We appreciate your valuable insight and have noted the gaps in the manuscript's limitations section.

2. Drug wastage and shortage are important topics, even more so since the COVID-19 pandemic. Reducing drug waste is an important step to help reduce drug shortages. The idea of monitoring drug usage and waste at the institution level and implementing surveys to monitor it. One aspect that we cannot control is safety. Sharing vials between patients, etc., should not be promoted, as it increases the risk of adverse events.

Author response: Again, we want to thank you for your contributions. We have addressed what was suggested and made the necessary adjustments by going over the supporting data in the manuscript's "split dose" and limitation sections.

3. Financial aspects are important, but it is a more complex topic. We could argue that cost-conscious care should be promoted. For example, in many countries, sugammadex has become the NMB reversal of choice despite the massive cost of the drug. The same is true for the routine use of dexmedetomidine, fibrinogen concentrate, etc. One should promote cost-conscious care in order to reduce the cost associated with the use of expansive drugs while cheaper and comparable drugs are available. Is there any data available out there in terms of cost-effectiveness for anesthetic drugs? I would suggest the authors address that.

Author response: We appreciate your input but respectfully decline to include its details in the manuscript for the following reasons:

1. Our review is specifically focused on identifying the prevalence and potential preventive strategies for anesthetic drug waste. Discussing other issues related to operating room efficiency, including cost-conscious anesthesia care, would be outside the scope and title of our review.
1. The publications included in our review do not consider cost-conscious anesthesia care as a preventive strategy for drug waste or as a method for increasing operating room efficiency.

However, we acknowledged the importance of cost-conscious anesthesia care at the beginning of the discussion and have highlighted.

4. I don't know if the conclusion is truly supported by the results of the systematic review. It is true, but there is an important gap in the literature.

Author response: Thank you once more for your contributions. We have rewritten the conclusion to integrate the gaps that readers should consider when applying preventive strategies.

5. As one of my colleagues would argue, the cost of anesthetic drugs only accounts for a small proportion of the overall cost when taking into account surgery, surgical tools, operating room time, ICU costs, etc.

Author response: We completely embraced your suggestion, which is why we have included these issues in the limitations section of the review.

6. I don't think it's been published, but several institutions have saved millions by monitoring drug usage and waste.

Author response: That is accurate, and we have noted that while there are numerous primary studies and non-systematic reviews on the topic, ours represents the first systematic review.

7. The discussion is interesting, but there are a lot of other aspects not discussed here.

Author response: We acknowledge the significance of your feedback, and as per your suggestions, we have integrated considerations such as scientific discussions on vial sharing and an emphasis on cost-conscious care.

8. It would be good if the authors could add a central figure or infographic summarizing the topic, their findings, and their suggestions.

Author response: Dear reviewer, We appreciate your feedback and recognize its importance. However, we believe that Table 2 adequately addresses this question by summarizing the topics covered in each study, along with their findings and suggestions. Introducing an additional table with related information may be redundant. Nevertheless, if you believe it is necessary to create another table to succinctly summarize the key points raised, we are prepared to do so.
